# Differentiation of Synanthropic Fleas from Andalusia (Spain) through Geometric Morphometrics Analysis

**DOI:** 10.3390/ani14111582

**Published:** 2024-05-27

**Authors:** Angela M. García-Sánchez, Ignacio Trujillo, Antonio Zurita, Cristina Cutillas

**Affiliations:** Department of Microbiology and Parasitology, Faculty of Pharmacy, University of Seville, Profesor García González 2, 41012 Sevilla, Spain; agarcia77@us.es (A.M.G.-S.); nachotr95@hotmail.com (I.T.); cutillas@us.es (C.C.)

**Keywords:** Siphonaptera, *Ctenocephalides*, *Pulex*, *Archaeopsylla*, morphometrics

## Abstract

**Simple Summary:**

Fleas are blood-sucking insects that are not only a nuisance but can also act as vectors for various diseases in animals and humans, including dangerous ailments such as the bubonic plague. Identifying and classifying these insects accurately is crucial for understanding how they spread and how to control them. Geometric morphometrics, a cutting-edge technique, is proving to be an invaluable tool in this regard, alongside traditional methods and molecular biology. In the present study conducted in Andalusia, Spain, this technique successfully differentiated between three populations of fleas, providing insights into their distribution, size, and characteristics. Image processing software was employed to obtain measurements, such as perimeters and areas, of the fleas under study. These findings underscore the importance of geometric morphometrics in studying and managing arthropod populations, particularly in cases where other methods fall short or are not available.

**Abstract:**

Fleas (Siphonaptera) are ectoparasitic hematophagous insects responsible for causing bites and itchy skin conditions in both humans and animals. Furthermore, they can act as vectors of different pathogens of a wide variety of diseases worldwide, including bartonellosis, rickettsiosis, and bubonic plague. Accurate identification of fleas is necessary for the study of their epidemiology, prevention, and control. In addition to traditional morphological classification approaches and molecular biology techniques, geometric morphometrics is increasingly proving to be a useful complementary tool for discriminating between Siphonaptera taxa. With the objective of determining the capacity of this technique to identify and differentiate synanthropic fleas, a principal component analysis was carried out on populations of *Ctenocephalides felis*, *Pulex irritans*, and *Archaeopsylla erinacei* collected in distinct regions of Andalusia (Spain). The analysis carried out on 81 male and female specimens revealed factorial maps that allowed the differentiation of the populations under study, with only partial overlaps that did not prevent their correct identification. Global size differences were also detected, with a slightly larger size in *P. irritans* males and a bigger size in *A. erinacei* females. Therefore, the present study emphasizes the role of geometric morphometrics as a useful complementary technique in taxonomic studies of arthropods, especially in the case of flea specimens lacking representative morphological features.

## 1. Introduction

Fleas (Siphonaptera) comprise a highly specialized order of holometabolous ectoparasitic insects with a cosmopolitan distribution and about 2700 species described so far [1]. In addition to being able to provoke bites and pruritic welts on the skin, these arthropods are also known to be vectors of different pathogens, responsible for causing a wide variety of diseases worldwide, including bartonellosis, rickettsiosis, and bubonic plague [2,3,4,5,6,7,8,9]. This is due to the nature of some flea species, which present a low host specificity that facilitates the exchanging of microorganisms, posing a potential threat to the health of both humans and animals [10,11,12].

The prevention and control of fleas require a large investment of money per year, which represents a significant economic burden [13]. It is crucial to enhance our knowledge about the taxonomy of fleas to develop effective strategies to reduce flea infestations and their negative impact on our environment.

Recently, in addition to the traditional morphological identification and molecular biology approaches, geometric morphometrics has proven to be a useful complementary technique for discriminating taxa across different groups [14,15,16]. One of the main features of geometric morphometrics is that it is especially helpful in cases of taxa that present morphological ambiguity [15], a situation relatively common in fleas [17,18,19]. This scenario invited the exploration of the affordable criterion offered by geometric morphometrics in systematic studies on flea genera, with promising results in *Ctenocephalides* Stiles & Collins, 1930 [20,21], *Ctenophthalmus* Kolenati, 1856 [22], *Pulex* Linnaeus, 1758 [23], and *Stenoponia* Jordan & Rothschild, 1911 [24].

In Europe, there is evidence of an escalating frequency of vector-borne diseases and heightened pathogen circulation, primarily influenced by human-related factors [25]. One region remarkably affected by arthropod-borne diseases is Andalusia, situated in the southern of Spain, where the West Nile Virus circulation is more widespread than initially considered [26], and outbreaks have been reported in recent years [27].

Furthermore, the incidence of murine typhus, a zoonosis caused by *Rickettsia typhi* da Rocha Lima, 1916 transmitted to humans by fleas, seems to be increasing slowly in Andalusia [28], an aspect that reveals the ability of some flea species present in the region to transmit pathogenic bacteria. Hence, it is essential to resort to techniques that allow us to safely discern between taxa. 

The cat flea, *Ctenocephalides felis* Bouché, 1835, succeeded in its expansion as a global parasite, and it is one of the most common flea species identified in domestic dogs and cats worldwide [11,19]. In Southwestern Europe, *C. felis* is the dominant species, although, in Eastern Europe, the infestation by *Ctenocephalides canis* Curtis, 1826 and *Pulex irritans* Linnaeus, 1758 also occurs [19,29,30,31].

On the other hand, hedgehogs inhabit rural, urban, and suburban environments, and they are frequently parasitized by blood-sucking arthropods, such as hard ticks and fleas, including the hedgehog flea, *Archaeopsylla erinacei* Bouché, 1835, and other flea species such as *C. felis*, *C. canis*, and *Nosopsyllus fasciatus* Bosc, 1800 [32,33,34,35]. Since they usually cohabit with pets and humans, they can potentially act as reservoirs of pathogen microorganisms responsible for zoonoses [12].

The main objective of the present study was to determine the capacity of geometric morphometric analysis to identify and discriminate fleas from populations of *C. felis*, *P. irritans*, and *A. erinacei* collected in Andalusia, in order to strengthen its role as a useful complementary technique in arthropod taxonomical studies.

## 2. Materials and Methods

### 2.1. Collection of Samples

Over a period of 19 months, we collected flea samples from dogs (*Canis lupus familiaris* Linnaeus, 1758) and one hedgehog (*Erinaceus europaeus* Linnaeus, 1758) that coexisted with other dogs. 

To gather flea samples from the hosts, we reached out to some veterinary clinics, veterinary hospitals, pet shelters, and some pet owners. In total, we contacted 145 veterinary clinics and 30 pet shelters and kennels. Among these, 18 centers agreed to participate in the sample collection (see Acknowledgements). All participants volunteered for this sampling process. Only animals parasitized by fleas were sampled. Veterinary practitioners performed an initial inspection of pets brought to their facilities. Each pet was checked for fleas and examined by a veterinarian who recorded clinical signs related to flea infestation. Adult flea counts were conducted according to the World Association for the Advancement of Veterinary Parasitology guidelines [36]. In brief, the animals were combed over their entire bodies with a fine-toothed comb for 5–10 min. 

All captured fleas from each infested host were transferred to a plastic 1.5 mL tube containing 96% ethanol for subsequent identification and morphometrics analyses.

### 2.2. Morphological Identification and Metric Data Processing

For morphological analysis, all specimens were initially examined under an optical microscope for specific classification. Following this, the specimens were cleared using 10% KOH, prepared, and mounted on glass slides following conventional procedures with the EUKITT mounting medium (O. Kindler GmbH & Co., Freiburg, Germany) [37]. The cleared and mounted specimens were examined again for a more detailed morphological analysis using a BX61 microscope (Olympus, Tokyo, Japan) and submitted to image capture processes with the imaging software cellSens Standard version 4.2 (Olympus, Tokyo, Japan). The diagnostic morphological characters of all samples were analyzed by comparison with figures, keys, and descriptions reported previously [38,39,40,41,42]. The measurement images of each flea were made using the image analysis software Image-PRO v11 (Media Cybernetics, Rockville, MD, USA). A total of 28 different parameters were measured for males and 36 for females (Table 1 and Table 2).

Descriptive univariate statistics based on arithmetic mean, standard deviation, and coefficient of variation for all parameters were determined for male and female populations. The data were subjected to one-way ANOVA (analysis of variance) for statistical analysis of the parameters. The results were statistically significant when *p* < 0.05. Statistical analysis was performed using Microsoft Excel for Microsoft 365 MSO (v2402). In addition, biometric characters of fleas were compared between different species and the most significant parameters were assayed for a morphometrics study. 

Morphological variation was quantified using geometric morphometrics [43], a technique that provides an estimate of size integrating different growth axes into a single variable known as “centroid size” [44]. The estimate of size was represented by a single variable that reflected variation in multiple directions, as many as there were landmarks under study, and shape was defined as their relative positions after correction for size, position, and orientation. With these informative data, and the corresponding software freely available to conduct complex analyses, significant biological and epidemiological features can be quantified more accurately [45]. 

Multivariate analyses were applied to assess phenotypic variations among the samples, using size-free canonical discriminant analysis on the covariance of log-transformed measurements. These analyses are applied to exclude the effect of within-group ontogenetic variations by reducing the effect of each character on the first pooled within-group principal component (a multivariate size estimator) [46]. principal component analysis (PCA) was used to summarize most of the variations in a multivariate dataset in a few dimensions [47]. Morphometric data were explored using multivariate analysis in three parameters in males (TW, HW, and AW) (Table 1) and females (Global Circularity, BULGAP, and HL) in females (Table 2) using BAC v.2 software [21,48].

## 3. Results

A total of 81 fleas (34 males and 47 females) were collected from different regions of Andalusia and classified as follows: 39 as *C. felis* (18 males and 21 females), 19 as *P. irritans* (6 males and 13 females), and 23 as *A. erinacei* (10 males and 13 females) (Table 3). All *C. felis* specimens and three *P. irritans* (the only male from Huelva and two female fleas from Seville) were collected from dogs (*Canis lupus familiaris*), whereas *A. erinacei* were collected from a hedgehog host. The rest of the *P. irritans* fleas were collected off-host from a neglected horse stable (Table 3). 

**Figure 1:**
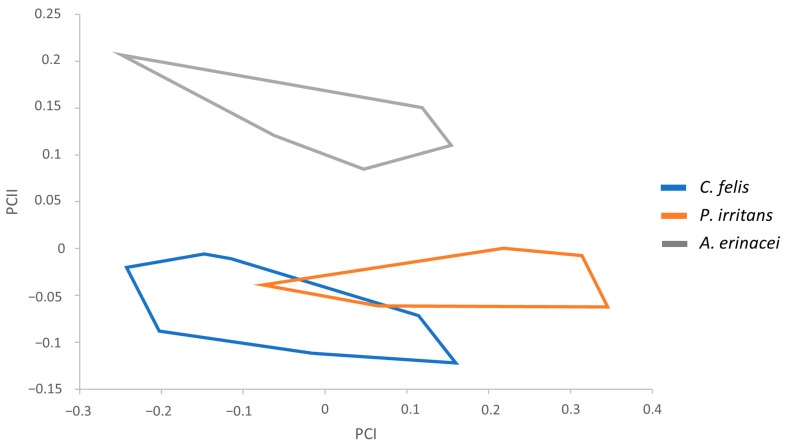
Factor map corresponding to *C. felis*, *P. irritans*, and *A. erinacei* male adults. Samples are projected onto the first and second principal components: PC1 (71%) and PC2 (26%). Each group is represented by its perimeter.

**Figure 2:**
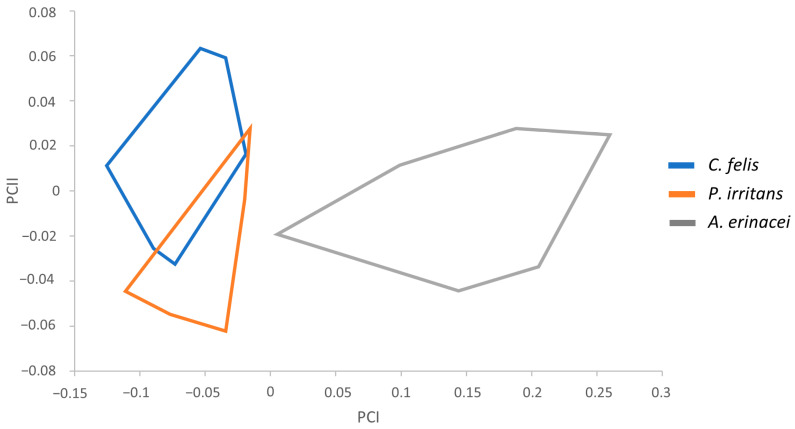
Factor map corresponding to *C. felis*, *P. irritans*, and *A. erinacei* female adults. Samples are projected onto the first and second principal components: PC1 (90%) and PC2 (8%). Each group is represented by its perimeter.

To carry out the classification of the samples, we considered descriptions used traditionally to discern between these species and, additionally, remarkable morphological features based on the measurements performed. Statistical tests showed several significant measurements for subsequent morphometric analyses. Therefore, the following parameters were used: total width (TW), total width of the head (HW), and apex width (AW) in males (Table 1) and total length of the head (HL), perimeter of the bulga (BULGAP), and Global Circularity in females (Table 2). This perimeter is the length of the bulga’s boundary, whereas the circularity is calculated as the ratio of the area of an object against a circle whose diameter is equal to the object’s maximum feret. The influence of size was analyzed using PCA in *C. felis*, *P. irritans*, and *A. erinacei*, involving the regression of each character separately on the within-group first principal component (PC1). The resulting factor maps for male and female populations are shown in Figure 1 and Figure 2, respectively.

Male variables significantly correlated with PC1, contributing 71% to the overall variation. The male factor maps showed global size differences in the flea populations, with a slightly larger size in *P. irritans* males (Figure 1). The three male communities are well grouped in the factor map, with a lack of noteworthy overlapping areas between them. Only *C. felis* and *P. irritans* showed a partial overlap but with no inconvenience in their identification.

On the other hand, female variables significantly correlated with PC1, contributing 90% to the overall variation. The resulting factor maps (Figure 2) clearly illustrate global size differences in the populations analyzed, including a bigger size in *A. erinacei*. As in the previous factor maps, there is a lack of notable overlapping areas between the female populations. As in the male factor maps, *C. felis* and *P. irritans* showed a partial overlap that did not prevent their identification.

## 4. Discussion

The accurate classification of fleas requires careful morphological examination or molecular confirmation, and therefore, the possibility that prior studies may have inadvertently misidentified fleas cannot be discarded [19,49]. In fact, authors like Ménier and Beaucournu reported numerous misidentifications in specimens of the genus *Ctenocephalides* [50]. The study of flea epidemiology, control, and prevention requires the accurate identification of species and subspecies.

In general, the classification of genera and species of fleas is based on external morphological characters. The presence or absence of combs and eyes, along with the length of the head, are typically significant features in morphological identification [51]. However, the size itself could never represent a way to reliably recognize the sex or the species of a flea specimen [52], and some flea species do not have easily identifiable morphological characters. For instance, *A. erinacei* and *P. irritans* do not possess pronotal or genal combs, so their classification process can be more complex in case the required specialized skills in flea identification are lacking. It is necessary to pay great attention to detail to recognize developmental stages and adequate sex identification [52]. Additionally, *C. felis* is a good example of a flea species known for its morphological ambiguity and the underlying issues in the study of their global populations [19]. In terms of molecular biology techniques, the notable lack of large-scale phylogenetic data for flea taxa causes some genera like *Ctenocephalides* to not have a defined genetic identity [17,19,40], especially if we consider subspecies [19].

Furthermore, it is still surprising that, despite the considerable veterinary and public health significance of dog fleas, studies investigating the diversity of these ectoparasite species on pets and the occurrence of flea-borne pathogens are scarce in certain regions [20].

Given the difficulties associated with flea morphological identification, the limited genetic information available, and the insufficient knowledge of the common pathogens of each flea species, the need to resort to complementary diagnosis techniques arises.

Geometric morphometrics analysis is one of these novel approaches applied to parasitological diagnosis, usually employed in arthropod identification [43]. The technique is based on the utilization of computer software for data processing and interpretation, with the advantage being that costly reagents and equipment are not required. Its affordability and the simplicity of data collection make it especially useful in low-resource settings [52,53].

To the best of our knowledge, the present work is the first in which flea measurements have been obtained using the imaging software Image-PRO. This program has been used before to study arthropods and other parasites [54,55,56,57]. It allowed us to incorporate into the analysis accurate measures such as areas, perimeters and circularities of the flea specimens for the first time. Global Circularity and BULGAP were revealed as useful features that contribute to the identification of flea species via geometric morphometrics.

The distribution of the three analyzed flea species showed a comparable pattern in both factor maps. *A. erinacei* appeared distant from *C. felis* and *P. irritans*, showing an appreciably larger size in the case of females. *C. felis* and *P. irritans* presented a small overlapping area, which did not prevent their individual identification. In both cases, *P. irritans* has always appeared larger than *C. felis*.

Although *A. erinacei* and *C. felis* are part of the Pulicinae family, the factor maps illustrated that fleas at the same taxonomic level are not necessarily closer at the morphological level since *A. erinacei* appears further away from both *C. felis* and *P. irritans*.

The selection of representative measurements for the morphometrics analyses was in accordance with previously published works. Total width (TW), head width (HW), and apex width (AW) are consolidated as useful parameters that define the morphological identity in males [21,50,58], as well as the total length of the head (HL) and the perimeter of the bulga (BULGAP) for females [21]. Due to the lack of genal ctenidium in *P. irritans* and *A. erinacei*, the difference in length between first and second spines (DEG parameter [21]) could not be considered in the present analysis, whereas the inclusion of the degree of elongation of the apical part (hilla) in females offered similar results but with a bit more overlap between *P. irritans* and *C. felis*. This is why the parameter APEHILL [21] was substituted by BULGAP, which permits the best differentiation between species.

The three analyzed flea species are among the most frequent in our environment, and, as a result, there is a notable risk of encountering them, with consequently associated parasitism suffered by humans and animals. After applying geometric morphometrics to differentiate flea communities of the same genera [21,22,23,24], the present work represents a step further, since this technique allowed us to identify different flea genera.

## 5. Conclusions

Accurate identification of fleas is necessary for studying the epidemiology, prevention, and control of these arthropods. In situations of uncertainty, alternative approaches are required to ensure correct classification. Geometric morphometrics is increasingly recognized as a reliable complementary technique for identifying flea species, particularly valuable in environments with limited resources.

In the present work, we were able to discern between the flea species *A. erinacei*, *P. irritans*, and *C. felis* using principal component analysis of males and females. Differences in overall size were also detected: *A. erinacei* presented the largest size in females, whereas *P. irritans* was slightly larger in males. Therefore, morphometrics is a relevant technique with great potential for application in the field of fleas, considering the existence of species that have traditionally posed challenges in their identification.

## Figures and Tables

**Table 1:** Biometrical data of males of *C. felis*, *P. irritans*, and *A. erinacei* collected from *Canis lupus familiaris* and *Erinaceus europaeus* from Andalucia (Spain).

	*C. felis*					*P. irritans*					*A. erinacei*				
	MAX	MIN	Mean	SD	VC	MAX	MIN	Mean	SD	VC	MAX	MIN	Mean	SD	VC
Global measures															
Area (mm^2^) †	1058.4	659.3	847.3	123.4	15	1379.8	688.6	1009.6	276	27	1436.7	990.3	1225.5	153.3	13
Roundness †	2.41	2.02	2.20	0.11	5	2.06	1.88	1.95	0.08	4	2.48	2.09	2.32	0.14	6
Circularity †	0.36	0.30	0.33	0.02	5	0.5	0.42	0.46	0.03	6	0.43	0.35	0.38	0.02	6
Perimeter (µm) †	5642.4	4213.9	4828.5	418.3	9	5676.4	4114.8	4925.4	649.5	13	6685.8	5424.4	5958	410.7	7
TL (µm)	2084	1563.0	1777.7	138.3	8	2027.7	1312.2	1650.0	285.8	17	2094.5	1667.3	1877.9	158.5	8
TW (µm) †	815.1	627.5	722.1	52.6	7	1064.1	772.3	926.0	120.9	13	989.4	810.1	890.0	56.4	6
Head measures															
Area (µm^2^) †	78,287	53,982	68,194	6318	9	100,673	74,318	83,921	9520	11	145,712	114,111	127,334	11,327	9
Roundness †	1.39	1.29	1.35	0.02	2	1.31	1.23	1.27	0.03	2	1.24	1.17	1.19	0.02	2
Circularity †	0.46	0.42	0.44	0.01	3	0.50	0.45	0.48	0.02	4	0.63	0.59	0.61	0.02	3
Perimeter (µm) †	1153	953.8	1072.6	49.8	5	1261.0	1071.6	1153.9	68.6	6	1502.9	1302.7	1379.8	64.0	5
HL (μm) †	391.1	306.8	360	22.9	6	357.0	293.4	317.6	25.0	8	453.2	378.3	412.6	23.2	6
HW (μm) †	251.2	210.0	228.4	12.1	5	263.0	228.3	248.2	15.1	6	378.1	323.4	352.2	16.2	5
Prothorax measures															
Area (µm^2^) †	24,848	14,287	19,192	3395	18	20,140	9635	13,081	3712	28	33,268	18,711	28,802	4038	14
Roundness †	1.45	1.23	1.37	0.06	4	2.08	1.68	1.90	0.14	7	1.60	1.25	1.43	0.11	8
Circularity †	0.58	0.46	0.51	0.04	8	0.36	0.26	0.32	0.04	11	0.61	0.38	0.46	0.07	16
Perimeter (µm) †	665.5	478.9	570.2	50.3	9	651.1	466.6	550.1	60.4	11	778.0	594.9	715.2	53.0	7
PROTW (μm) †	120.4	82.0	100.8	11.0	11	83.5	46.1	60.1	12.8	21	151.0	88.6	120.7	16.6	14
Mesothorax measures															
Area (µm^2^) †	31,142	14,043	22,358	4518	20	28,396	8581	18,186	7347	40	33,772	23,289	28,643	3716	13
Roundness †	1.64	1.33	1.46	0.08	6	2.05	1.65	1.81	0.19	10	1.92	1.40	1.72	0.19	11
Circularity †	0.57	0.42	0.48	0.04	8	0.43	0.29	0.37	0.05	15	0.55	0.37	0.42	0.05	13
Perimeter (µm) †	723.9	512.8	634.1	63.3	10	764.6	467.7	624.0	107.4	17	858.8	670.8	783.0	64.7	8
MESOW (μm) †	137.4	88.7	109.0	13.8	13	103.5	47.7	76.2	21.8	29	137.0	99.3	111.3	13.6	12
Metathorax measures															
Area (µm^2^) †	37,152	20,305	28,528	5061	18	53,522	20,280	35,569	12,210	34	48,486	33,659	38,673	4209	11
Roundness †	1.57	1.28	1.46	0.07	5	1.64	1.38	1.53	0.10	7	1.81	1.47	1.60	0.10	7
Circularity †	0.57	0.39	0.44	0.04	10	0.47	0.42	0.44	0.02	5	0.42	0.34	0.39	0.02	6
Perimeter (µm) †	877.8	625.5	758.6	71.6	9	1024.4	667.7	857.8	123.5	14	1023.2	883.7	922.7	41.9	5
METW (μm) †	142.8	102.3	120.4	12.6	10	177.1	97.5	131.4	30.4	23	157.1	109.7	134.2	13.9	10
AW (μm) †	34.9	12.9	20.7	6.0	29	51.3	19.2	37.0	12.2	33	31.3	11.3	23.1	6.2	27

TL = total length, TW = total width, HL = total length of the head, HW = total width of the head, PROTW = total width of the prothorax, MESOW = total width of the mesothorax, METW = total width of the metathorax, AW: apex width, MAX = maximum, MIN = minimum, SD = standard deviation, Mean = arithmetic mean, VC = coefficient of variation (percentage converted), † = significant differences between groups (*p* < 0.005).

**Table 2:** Biometrical data of females of *C. felis*, *P. irritans*, and *A. erinacei* collected from *Canis lupus familiaris* and *Erinaceus europaeus* from Andalucia (Spain).

	*C. felis*					*P. irritans*					*A. erinacei*				
	MAX	MIN	Mean	SD	VC	MAX	MIN	Mean	SD	VC	MAX	MIN	Mean	SD	VC
Global measures															
Area (mm^2^)	2290.4	827.8	1724.7	438.2	25	2876.6	1180.3	1596.4	567.1	36	2874.6	1368.8	2023.6	434.1	21
Roundness †	2.57	1.82	2.10	0.20	10	2.11	1.80	1.90	0.08	4	2.34	1.76	2.02	0.17	8
Circularity †	0.40	0.33	0.36	0.02	5	0.50	0.40	0.46	0.02	5	0.49	0.36	0.41	0.03	8
Perimeter (µm) †	7967.9	4583.8	6661.7	837.0	13	8379.8	5310.4	6092.9	993.7	16	8733.5	6038.3	7127.7	902.6	13
TL (mm) †	2859.5	1616.2	2439.8	337.8	14	2861.0	1757.8	2041.3	372.8	18	2938.3	1885.7	2425.1	332.8	14
TW (mm) †	1208.6	767.4	1038.7	136.2	13	1495.9	1003.5	1150.2	144.0	13	1487.5	1058.3	1220.5	112.7	9
Head measures															
Area (µm^2^) †	100,169	67,749	83,173	9264	11	135,023	87,601	111,137	12,669	11	163,067	107,663	135,800	18,243	13
Roundness †	1.55	1.35	1.45	0.05	3	1.40	1.22	1.30	0.06	4	1.26	1.16	1.20	0.02	2
Circularity †	0.46	0.38	0.42	0.02	5	0.50	0.43	0.46	0.02	5	0.58	0.51	0.55	0.02	4
Perimeter (µm) †	1354.0	1102.8	1229.3	73.2	6	1455.0	1238.6	1342.4	65.3	5	1564.9	1273.1	1423.1	89.7	6
HL (μm) †	449.5	357.9	406.6	27.3	7	428.5	344.0	371.5	24.4	7	463.8	380.5	421.3	26.4	6
HW (μm) †	296.8	243.2	265.4	15.1	6	330.1	243.7	287.6	22.2	8	383.9	274.2	324.7	30.5	9
Prothorax measures															
Area (µm^2^) †	36,972	15,844	25,774	5347	21	29,118	16,764	22,842	3602	16	48,287	25,729	37,136	7542	20
Roundness †	1.92	1.40	1.65	0.14	8	2.22	1.53	1.78	0.18	10	1.50	1.26	1.42	0.08	5
Circularity †	0.53	0.36	0.45	0.05	10	0.43	0.26	0.34	0.04	13	0.59	0.41	0.48	0.05	11
Perimeter (µm) †	876.7	576.8	723.8	74.3	10	812.5	615.0	709.3	51.4	7	879.0	691.0	805.6	71.8	9
PROTW (μm) †	139.0	67.7	105.0	18.3	17	98.6	56.9	84.8	12.2	14	168.1	106.3	138.2	20.2	15
Mesothorax measures															
Area (µm^2^)	48,419	20,032	35,002	8398	24	49,243	27,112	37,914	5734	15	50,800	29,397	40,732	6543	16
Roundness †	1.69	1.24	1.51	0.11	7	1.86	1.52	1.64	0.10	6	1.86	1.50	1.60	0.12	7
Circularity †	0.60	0.33	0.46	0.06	13	0.46	0.35	0.42	0.03	7	0.51	0.40	0.47	0.03	6
Perimeter (µm) †	967.0	638.1	807.6	101.4	13	1031.0	732.8	879.5	78.2	9	1012.9	783.6	899.3	75.2	8
MESOW (μm) †	161.4	88.1	135.2	19.0	14	145.5	105.9	124.5	10.0	8	168.1	113.8	140.8	15.6	11
Metathorax measures															
Area (µm^2^) †	63,421	26,207	46,240	10,448	23	73,621	48,603	63,117	7854	12	66,054	44,555	54,053	7425	14
Roundness †	2.09	1.41	1.68	0.17	10	1.73	1.37	1.51	0.10	7	1.59	1.31	1.48	0.07	5
Circularity †	0.48	0.34	0.41	0.03	9	0.48	0.37	0.44	0.03	8	0.50	0.40	0.44	0.03	7
Perimeter (µm) †	1185.4	772.5	979.9	128.9	13	1199.2	943.2	1090.1	70.3	6	1116.9	897.5	997.9	73.2	7
METW (μm) †	175.8	109.0	147.5	18.5	13	206.9	145.3	178.4	15.8	9	188.3	144.9	170.2	16.1	9
Spermatheca measures															
Area (µm^2^) †	3088	2162	2619	259	10	3489	2439	3062	309	10	11,768	3875	7474	2120	28
Roundness †	1.21	1.05	1.14	0.04	4	1.06	1.01	1.03	0.01	1	1.25	1.05	1.12	0.06	6
Circularity †	0.76	0.59	0.65	0.05	7	0.89	0.76	0.85	0.04	5	0.81	0.53	0.66	0.08	12
BULGAP (µm) †	212.1	167.8	192.7	10.9	6	210.8	176.3	198.0	10.3	5	396.7	227.0	320.4	45.6	14
BULGAL (μm) †	71.5	52.6	61.2	5.0	8	70.6	54.3	63.9	4.7	7	127.0	80.3	99.7	15.0	15
BULGAW (μm) †	58.2	46.4	50.9	3.0	6	66.7	55.6	59.6	3.1	5	133.7	57.8	90.8	22.6	25
APEHILL (μm) †	52.8	22.9	35.6	9.1	25	64.2	26.9	46.9	12.2	26	118.9	38.4	83.4	21.0	25
APEHILW (μm) †	31.7	17.0	24.7	4.4	18	64.5	23.4	31.7	10.7	34	57.9	20.9	38.1	9.4	25
DBMV (μm) †	349	131	251	57.1	23	485	290	378	56.7	15	503.8	150.3	293.4	116.5	40

TL = total length, TW = total width, HL = total length of the head, HW = total width of the head, PROTW = total width of the prothorax, MESOW = total width of the mesothorax, METW = total width of the metathorax, BULGAP: perimeter of the bulga, BULGAL: total length of the bulga, BULGAW: total width of the bulga, APEHILL: total length of the apex of the hilla, APEHILW: total width of the apex of the hilla, DBMV = distance from bulga to the ventral margin of the body, MAX = maximum, MIN = minimum, SD = standard deviation, Mean = arithmetic mean, VC = coefficient of variation (percentage converted), † = significant differences between groups (*p* < 0.005).

**Table 3:** Distribution of fleas collected from dogs from different geographical origins.

Geographical Origin	*C. felis* (Number of Fleas)		*P. irritans* (Number of Fleas)		*A. erinaceid* (Number of Fleas)	
	Male	Female	Male	Female	Male	Female
Sanlúcar de Barrameda (Cadiz, Spain)	18	21	-	-	-	-
Seville (Seville, Spain)	-	-	5	13	-	-
Huelva (Huelva, Spain)	-	-	1	-	-	-
Dos Hermanas (Seville, Spain)	-	-	-	-	10	13
Total	18	21	6	13	10	13

## Data Availability

The data presented in this study are available in the article.
